# Depicting the interplay between organisational tiers in the use of a national quality registry to develop quality of care in Sweden

**DOI:** 10.1186/s12913-015-1188-2

**Published:** 2015-11-25

**Authors:** Ann Catrine Eldh, Mio Fredriksson, Sofie Vengberg, Christina Halford, Lars Wallin, Tobias Dahlström, Ulrika Winblad

**Affiliations:** Department of Public Health and Caring Sciences, Uppsala University, Box 564, SE751 22 Uppsala, Sweden; School of Health and Social Science, Dalarna University, SE791 88 Falun, Sweden; Division of Nursing, Department of Neurobiology, Care Sciences and Society, Karolinska Institutet, SE171 77 Stockholm, Sweden

**Keywords:** Clinical database, National quality registry, Implementation, Quality improvement, Medical registry

## Abstract

**Background:**

With a pending need to identify potential means to improved quality of care, national quality registries (NQRs) are identified as a promising route. Yet, there is limited evidence with regards to what hinders and facilitates the NQR innovation, what signifies the contexts in which NQRs are applied and drive quality improvement. Supposedly, barriers and facilitators to NQR-driven quality improvement may be found in the healthcare context, in the politico-administrative context, as well as with an NQR itself. In this study, we investigated the potential variation with regards to if and how an NQR was applied by decision-makers and users in regions and clinical settings. The aim was to depict the interplay between the clinical and the politico-administrative tiers in the use of NQRs to develop quality of care, examining an established registry on stroke care as a case study.

**Methods:**

We interviewed 44 individuals representing the clinical and the politico-administrative settings of 4 out of 21 regions strategically chosen for including stroke units representing a variety of outcomes in the NQR on stroke (Riksstroke) and a variety of settings. The transcribed interviews were analysed by applying The Consolidated Framework for Implementation Research (CFIR).

**Results:**

In two regions, decision-makers and/or administrators had initiated healthcare process projects for stroke, engaging the health professionals in the local stroke units who contributed with, for example, local data from Riksstroke. The Riksstroke data was used for identifying improvement issues, for setting goals, and asserting that the stroke units achieved an equivalent standard of care and a certain level of quality of stroke care. Meanwhile, one region had more recently initiated such a project and the fourth region had no similar collaboration across tiers. Apart from these projects, there was limited joint communication across tiers and none that included all individuals and functions engaged in quality improvement with regards to stroke care.

**Conclusions:**

If NQRs are to provide for quality improvement and learning opportunities, advances must be made in the links between the structures and processes across all organisational tiers, including decision-makers, administrators and health professionals engaged in a particular healthcare process.

**Electronic supplementary material:**

The online version of this article (doi:10.1186/s12913-015-1188-2) contains supplementary material, which is available to authorized users.

## Background

Healthcare quality registries (also labelled medical registries, clinical databases and the like) denote standardised and complete sets of systematically collected and entered individualised data concerning patient problems, and healthcare interventions and outcomes. As such, quality registries have been identified as a promising route for improved quality of care [1]. Supposedly, registries can provide healthcare performance information, motivate change, spur quality improvement activities, and enable health professionals’ engagement in continuous learning through identifying and sharing best clinical practices [2, 3]. While data feedback from registries has been suggested as one of the most important factors for improved quality of care, it is also known that the quality of the data and the timeliness of the feedback is vital [4]. Further, the data itself and the feedback initiatives must correspond with the organisational context. The effectiveness of feedback is positively influenced by components such as tailored quality improvement initiatives performed by local teams [5]. Altogether, the success of quality improvement demands that this data feedback from medical registries is accompanied by local improvement efforts.

In order to support research and quality improvement, many countries have been influenced by the particular efforts of Sweden to develop and employ shared medical registries [6–8]. With a unique and reasonably long history of what are labelled National Quality Registries (NQRs), Sweden today has 81 NQRs and 24 NQR candidates, all of them corresponding to national criteria and thus receiving financial support by the government [9]. Furthermore, between 2012 and 2016, the NQRs have been awarded an additional investment by the government to increase the use of the NQRs in healthcare quality improvement, and to facilitate registry-based research [10]. The NQRs’ usefulness in research is rather well-documented (for example [3, 11]), whereas the understanding of how the NQRs contribute to local quality improvement is in its early stages.

When studying the link between NQRs and quality improvement, a confounding factor is the multi-faceted character of the registries. As such, the NQRs may be defined as *meta-interventions,* signifying numerous features that possibly affect quality, and an almost infinite number of possible combinations of these features, particularly in relation to the local contextual conditions [12]. As meta-interventions may fuel multiple, simultaneous changes in organisational processes and structures, studying such interventions requires recognition of the context at multiple levels of healthcare delivery [13]. Thus, to understand how the NQRs contribute to local care quality improvement, what takes place in the implementing clinics is important, as are the barriers and facilitators to implementation. Moreover, the political and administrative context that ultimately defines the conditions for both the clinical practice and implementation, must be considered [14].

Sweden and many other countries (for example the UK, Australia, and Canada) have comprehensive, tax-funded healthcare systems, governed by a multi-tier organisation. The Swedish regional authorities that are accountable for the funding and provision of healthcare (that is, the 21 regions, also called county councils) are self-governing. Thus, the regional authorities are, to a certain extent, unrestricted in constituting the provision of services and decision-making, as well as in how they attend to quality improvement issues. Further, the regional authorities are autonomous in how to implement the government’s initiative to increase the use of NQRs in healthcare improvement [10].

In earlier studies we have found that NQRs can support local quality improvement, yet participation in a registry did not in itself initiate this process [15]. The NQR data could fit and serve as valid and reliable prompts for what needed attention, but the quality improvement itself was generated by local healthcare professionals collaborating within and outside their local context, given that they had the resources. Further, the politico-administrative decision-makers and administrators were found lacking in manifest leadership engagement related to the NQR and the local quality improvement, both at regional and healthcare provider levels [16]. Taking these studies into account, there are aspects of the interplay between the politico-administrative and clinical levels within the regions which could constitute barriers and facilitators for quality improvement in relation to the meta-intervention NQRs. Hence, a variation with regards to if and how an NQR is implemented could potentially depend on the degree of collaboration between the politico-administrative level and the clinical level with regards to NQRs. The aim of this paper was to depict the interplay between the clinical and the politico-administrative tiers in the use of NQRs to develop quality of care, examining an established registry on stroke care as a case study.

## Methods

### Design

This qualitative study is part of a larger, mixed methods research program investigating the decisions and activities for NQRs to be used for, and generating, local quality improvement [17]. The Consolidated Framework for Implementation Research (CFIR) [13, 18] was used to identify influential contextual constructs at politico-administrative and clinical levels, and to help generalise findings and facilitate comparisons, and well as to illustrate a lack or presence of interplay between tiers, and the consequences of these.

### Setting and sample

Because the national stroke registry (*Riksstroke*) exemplifies an NQR with long endurance and high coverage, it was selected for this case study: Riksstroke was launched in 1994, and is one of the most extensive Swedish NQRs [19]. All 72 hospitals providing acute stroke care partake in the registry, reporting altogether 25,000 to 26,000 unique healthcare cases of stroke each year, including the acute phase of stroke, and follow-up at 3 and 12 months following the stroke incident for each patient [20]. The Riksstroke is governed by a steering committee (overseeing the quality of the registry, advising the registry manager on for example development, and suggesting and executing research studies based on the registry data), and a secretariat (providing daily maintenance of the registry, and supporting managers and staff in the hospitals).

Out of the 21 regions in Sweden, a strategic sample was drawn from 8 hospitals in 4 regions (labelled A, B, C, and D) representing different parts of the country, and all types of Swedish hospitals: university/teaching hospitals, county hospitals, and local hospitals. Further, we drew the sample from the Riksstroke’s annual follow-up of 2011, assuring that we included stroke units representing ‘high’, ‘moderate’, and ‘not reaching moderate’ results in terms of the target levels for quality of care (presented in Table 1).

**Table 1:** Riksstroke’ s target levels for stroke quality indicators [20]

Digit	Quality indicator	Target levels (proportion of stroke patients)
A	Coverage	Moderate 85 %, high 92 %
B	Follow-up 3 months post-stroke	Moderate 85 %, high 90 %
C	Being treated in stroke unit	Moderate 85 %, high 90 %
D	Non-stop admission to stroke unit or intensive care	Moderate 80 %, high 90 %
E	Swallow test performed	Moderate 90 %, high 95 %
F	Treated with reperfusion	Moderate 10 %, high 15 %
G	Time from arrival to hospital to thrombolysis start	Moderate 60 min., high 40 min.
H	Antithrombotic treatment (regardless of type) at discharge	Moderate 85 %, high 90 %
I	Anticoagulation treatment post embolic brain infarction, <80 years of age	Moderate 55 %, high 70 %
J	Hypertension treatment post-stroke	Moderate 70 %, high 80 %
K	Statin treatment post-brain-infarction	Moderate 65 %, high 75 %
L	Needs for help and support after discharge totally met	Moderate 60 %, high 75 %
M	Follow-up out-patient visit, with physician and/or registered nurse	Moderate 80 %, high 90 %

**Fig. 1:**
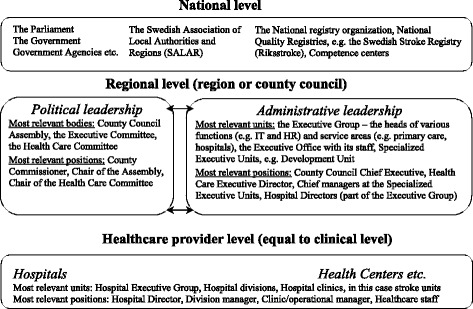
Chart of the Swedish healthcare system

For each region we included all organisational tiers relevant for the use of NQRs and the development of quality of care, including the politico-administrative and the clinical level of healthcare. An overall illustration of the Swedish healthcare system is presented in Fig. 1, providing a general illustration of regional and healthcare provider levels, respectively, in this study. In total, 44 individual interviews were performed: telephone interviews were chosen over face-to-face interviews to secure full flexibility in the interview schedule and availability of interviewers for the dates and time points suggested by the informants. At regional level, 19 interviews were performed with: county commissioners; healthcare executive directors; chief managers and co-workers of politico-administrative development units; and hospital directors. At healthcare provider level, 25 interviews were performed with: the head of the hospital division (if any) wherein the stroke unit was organised; the head of the hospital department where the stroke unit was managed; the physician in charge of the Riksstroke registry; and the registered nurse (RN) in charge of registering the local data for Riksstroke.

### Procedure

Representatives at the healthcare provider and the politico-administrative tiers, also called “stakeholders” in this study, were identified by initial contacts with hospitals and regions. After obtaining names and addresses, information about the study and an inquiry for an interview was sent via e-mail to each individual. A maximum of three reminders were sent and, if necessary, a final invitation was communicated via telephone. At the politico-administrative level, five stakeholders declined participation, but a substitute for each individual was identified. Thus, all regions in the sample were represented. At the healthcare provider level, only one manager declined participation, and no substitute was available.

Interviews were performed by four researchers, coordinated and guided by semi-structured interview guides developed for the case study. Questions included: 1) the informant’s role in relation to NQRs, and in particular, Riksstroke; 2) data capture and registration (for healthcare provider level only); 3) data reports, feedback, and data sharing; 4) quality improvement; 5) collaboration; 6) pros and cons of NQRs, in particular, Riksstroke; and 7) the usability and reliability of NQRs, in particular, Riksstroke. Additionally, different tiers’ work with NQRs and quality improvement was explored, along with supportive structures, including the issue of collaboration, and responsibilities and roles in terms of NQRs and quality improvement.

The interviews at the politico-administrative level lasted 30–60 minutes, and upper level management stakeholder interviews at healthcare provider level around 15 minutes. Further, interviews with clinical stakeholders (that is, healthcare provider level, including physicians and RNs involved in the local Riksstroke efforts) lasted 45–60 minutes. All interviews were recorded and transcribed verbatim. All participants gave informed consent to participation prior to the interviews, and approval of the study was obtained from the Research Ethics Board in Uppsala, Sweden (no. 2013/181).

### Data analysis

Applying deductive content analysis as described by Elo and Kyngäs [21], the initial phase included reading and re-reading the interview texts, bringing the understanding to “a sense of the whole”. Subsequently, data was categorised and abstracted with the CFIR as a coding framework. Similarly to Damschroder and Lowery [18], we relied on a ‘menu of constructs’ approach [22] which yielded flexibility in the employment of constructs – including that we applied only those constructs valid to the present study [18]. Consequently, prior to the structured analysis, the CFIR framework was scrutinised and discussed between the researchers with regards to the appropriateness of the domains and constructs and their correspondence with the data, that is, the interview texts at politico-administrative and healthcare provider levels. The application details of CFIR are presented in Additional file 1. In particular, references to interplay were investigated in the domains Inner Setting and Process. Three researchers were involved in the entire analysis process, repeatedly discussing aspects and outcomes to support its trustworthiness [23].

## Results

Applying the CFIR, we present the structured analysis of the ‘Intervention Characteristics’ (in this study representing NQRs such as the Riksstroke registry), which was found to depict one common representation across the four regions. However, the ‘Inner Setting’ (representing the politico-administrative, hospital, department, and stroke unit levels, including the structures and the healthcare process for stroke), as well as ‘Process’ (representing the quality improvement and the use of NQRs, and Riksstroke in particular, for this purpose) were found to vary between the regions. Thus, Inner Setting and Process, respectively, are presented in relation to the similarities and differences of the investigated regions. Throughout the findings, the CFIR constructs are indicated by italics. The CFIR domain Characteristics of Individuals was not used as the study did not focus on individual-level behaviour, and Outer Setting was found not to illustrate any differences or aspects that could illustrate the interplay and its influence on quality improvement by adopting NQRs, or Riksstroke in particular.

### Intervention characteristics

In this domain, we considered the quality registry to be the intervention.

Stakeholders at politico-administrative level tended to speak about NQRs in general, and were less familiar with the Riksstroke details, while the clinical stakeholders were knowledgeable about the Riksstroke in particular. To them, the NQR and its variables (corresponding to *evidence strength & quality*) were relevant to stroke care. Yet, with the multitude of variables and the need for all data to be entered by hand, registration was a meticulous and time-consuming process although it was perceived as being necessary for registering trustworthy data. All stakeholders demonstrated confidence in the data submitted by their hospitals’ stroke units. Further, the clinical stakeholders knew which other stroke units had the most valid reporting routines and considered those for their benchmark, besides monitoring their own stroke outcomes over time.

Stakeholders across the tiers considered Riksstroke and similar NQRs to have *relative advantages* over local medical registries. Advantages were primarily that the Riksstroke variables were based on the evidence-based national guidelines for stroke care, thus providing for comparisons against a reliable standard while tracking the local level of evidence-based practice. The fixed number of variables in Riksstroke left no room for local *adaptation* but adaptation occurred in how Riksstroke was applied towards quality improvement, as further described in the Process section below.

Because of *complexity* in terms of the number of NQRs and NQR variables, respectively, representatives at the politico-administrative in general, and healthcare provider managers to some extent, relied on the annual reports in the Regional Comparisons of quality and efficiency in Swedish HealthCare, rather than the NQRs, to capture the quality of care. Not only did the politico-administrative stakeholders not have direct access to the NQRs, but they preferred the Regional Comparisons with its selection of a number of variables from the NQRs and other registries. Further, the layout of the Regional Comparisons was considered appealing and understandable, overruling the fact that the further processing meant that the data were not as timely as the NQRs themselves and recognising that by awaiting the Regional Comparisons, they risked acting on outdated problems. At healthcare provider level, the RNs and physicians could instantly retrieve their own unit’s outdata in Riksstroke. Capturing the *design quality and packaging*, a barrier was often the time and commitment needed to perform these activities.

Even though Riksstroke and other NQRs originate from professional initiatives and perspectives, they were regarded by both decision-makers and administrators at politico-administrative and the professionals and managers at healthcare provider level as an administrative constraint and *cost*, and were perceived to be difficult to manage. An extensive amount of time and resources spent on the NQR was considered to be consumed mostly by data registration rather than outcome analysis and subsequent quality improvement initiatives.

### Inner setting

The domain, Inner Setting, constitutes the context at region and stroke unit level, as well as interactions between these tiers.

The *structural characteristics* differed between regions; while all stakeholders considered the NQRs an asset for local quality improvement, not all regions had structures and processes in place for the management of quality improvement using NQRs. Both regions A and B had initiated and performed large collaborative projects across tiers, securing a common stroke process for the hospitals within their regions. In region C, the politico-administrative management had recently initiated such efforts to better streamline the stroke care process. In regions A and B, clinical stakeholders had been engaged in these process projects led by politico-administrative representatives, and the politico-administrative representatives suggested this would be the case for region C as well. For region D, stakeholders at all tiers described a lack of proper structures for quality management, and limited contact between tiers. Rather, the stroke unit in the main hospital within region D had improved the stroke care process some years ago, without involving the politico-administrative leadership, who had little insight in terms of clinical quality improvement projects.

Further, the understanding of the structural characteristics for quality improvement within the stroke units was discussed in more detail by the physicians and RNs engaged in the local Riksstroke work compared to the politico-administrative stakeholders. More or less all healthcare provider level informants described the stroke care budgets as strained, and cited general difficulties in attracting and retaining medical and nursing staff specialised in stroke. While all eight stroke units in the four regions had allocated resources for the local Riksstroke work, the assignments of the clinical stakeholders varied. All clinical stakeholders described a responsibility for managing the registry and establishing high coverage, yet some also worked with compilation and *communication* of data to fellow staff and managers. Whether or not this was done depended on how much time they had set aside, the individual’s interest in stroke care, and the interest of their fellow staff and managers. Meanwhile, stakeholders at stroke units within the same regions described differences in terms of internal collaboration. Further, the collaboration with and the interest of the politico-administrative level for stroke care and the outcomes of Riksstroke varied.

Clinical stakeholders who collaborated within the stroke unit, and had opportunities to *feedback* tailored presentations of data to their managers and fellow staff, depicted the *culture* as nurturing. Nurturing conditions such as these existed across the regions, illustrating where the clinical stakeholder roles and responsibilities were well-defined, and communicated and recognised in the stroke units. Yet, the *implementation climate* was affected by the *tension* caused by the extensive data registration, influencing whether or not the registration was considered a *relative priority* compared to other clinical tasks and *compatible* with the stressful working conditions in the stroke units. Further implementation climate aspects illustrated that the politico-administrative stakeholders in the four regions were at different stages with regards to applying the data from Riksstroke. While they mainly accessed the stroke care outcomes through Regional Comparisons, some set stroke care targets and provided reimbursement (regions B and D), and identified issues to map and improve quality of care (regions A and B) by employing the NQR.

In regions A and B, *networks* and structures for *communications* were established, providing learning opportunities and dialogue for stakeholders across tiers. In regions C and D, stakeholders described no regional networks on stroke, yet the stroke process project in region C included opportunities to meet and discuss common issues. Yet, in region C, the health professionals engaged in Riksstroke depicted a limited engagement of other clinicians involved in stroke care and thus, these forums were not considered to be driving the local improvement.

The *culture* across tiers differed between regions. In A and B, where regional projects had been undertaken to improve the stroke care processes, the Riksstroke data were applied to identify issues in and between hospitals in the stroke process. Here, the decision-makers and administrators at politico-administrative level acknowledged the health professionals for their contribution but recognised their own responsibility for supporting the stroke process and for placing stroke care on the political agenda. The politicians were considered to be the primary suppliers of targets with regards to the healthcare provided for the citizens. In region C, the characteristics of the stroke care collaboration was not yet known, and in region D, there were no distinct interconnections between the politico-administrative and the healthcare provider levels.

The *readiness for implementation* (in this case, applying the NQR for quality improvement) was also found to vary: in regions A and B, stakeholders acknowledged the need for collaboration between tiers to improve the healthcare processes using NQRs such as Riksstroke. Meanwhile, in region C, the politico-administrative stakeholders suggested that the central management, at region level, should guide such efforts. The region D informants considered it a clinical issue which their hospital management was to oversee. Yet, region D administrators noted a current increase in *leadership engagement* among politicians in the NQR, and its potential role in assessing and promoting quality of care. While regions A, B, and C depicted having *available resources* in terms of staff for leading and coordinating NQR-related work at the politico-administrative level, region D had no such person or function at that level. All stroke unit RNs and physicians illustrated an *access to knowledge and information* through the Riksstroke itself whereas stroke unit and department managers relied on these clinicians to present the outdata. Only the clinicians at the stroke units who described that there was a mutual idea of stroke care being a top priority across tiers also depicted being empowered by the staff and *leadership engagement*.

### Process

For the domain Process, aspects of applying NQRs and the Riksstroke registry in particular for quality improvement were included, at politico-administrative and healthcare provider level and in between the tiers.

All stroke units across the regions had adopted the Riksstroke NQR long ago, and in some cases, a second generation of clinical stakeholders was involved. In regions A and B, stakeholders at both politico-administrative and healthcare provider level had *engaged* in *planning* and *executing* projects to manage the stroke care processes, applying the Riksstroke registry as an input. The clinical stakeholders retrieved outcome data for their stroke units and the Riksstroke data had been used for identifying improvement issues, for setting goals, and asserting that the stroke units achieved an equivalent standard of care and a certain quality of stroke care. Region C had hitherto just initiated a comparable project.

The extent to which the stakeholders retrieved and acted on the stroke data outcomes differed; whether to attend to NQR outcomes, which outcomes to focus on, and how to deal with the local quality issues detected, were independent decisions, varying from region to region and to some extent also from stroke unit to stroke unit. Regions A and B stakeholders depicted that the Riksstroke data had fed and enriched the stroke care process, and that the registry assisted in *evaluating and reflecting* on what was accomplished and what needed further attention. In region C, healthcare professionals *engaging* in the stroke process project were hindered by the lack of mandate to impact on the healthcare structure. Further, in region D, the process focused on what the responsible RN and physician did in terms of *monitoring* the clinic’s longitudinal development, which was regularly reported to the stroke unit’s staff, while the politico-administrative stakeholders were not engaged.

## Discussion

We found several aspects within the studied regions that differ in terms of how the NQRs, and in particular Riksstroke, were used for quality improvement and that the interactions between the tiers played a vital role in the process. More specifically, we argue that the key issues for quality improvement using NQRs were: a) stroke process development initiatives, including agreed goals for quality of care and healthcare production, b) structures and processes for mutual learning within the organisations, and c) leadership engagement.

The NQR Riksstroke was (and is) set to an agreed, general standard with the national guidelines on stroke being the main source of the variables [20]. Even though Riksstroke (and other NQRs) was originally formed by stroke experts [19], it is nowadays managed within a national framework [24]. This limits the local adaptability. Yet, it is highly flexible whether or not, and when and how to apply Riksstroke and other NQRs in quality improvement. We found that the regions where stakeholders described the most extensive use of Riksstroke also described the most extensive collaboration between tiers. In the two regions where stakeholders depicted a joint effort to an agreed stroke care process, they also illustrated the most communication across tiers with regards to the use of Riksstroke data. These joint efforts were initiated by the politico-administrative stakeholders, and included collaboration with the clinical stakeholders in the stroke units. Yet, clinical managers were not involved, even though other clinical stakeholders have described local collaboration with them as being crucial [15].

Although the local Riksstroke data were applied in the stroke process projects, continuous collaboration between the levels within the regions with regards to the NQR was limited. A cause depicted was that only the clinical stakeholders were knowledgeable about retrieving and interpreting data from Riksstroke, while the stakeholders at politico-administrative level relied on the reports presented by national bodies. Data feedback is suggested as one of the main advantages of quality registries, driving the quality improvement [9]. Yet, our findings illustrate that the accessibility and possibilities to comprehend data need to improve. Further, continuous opportunities to collaborate between tiers in the regions on what the data represent are needed, beyond particular projects. Increased collaboration between tiers could prevent the depicted risk that healthcare management decisions and priorities are based on outdated data, and that a lack of leadership engagement demotivates the clinical stakeholders [15]. Rather, communication across tiers and leadership engagement are both factors known to have a positive effect on organisational innovation [25].

The core components for quality improvement were the outcomes and goals of stroke care identified by using Riksstroke in the stroke care process development; two of the four regions in this study described using the Riksstroke for setting goals and reimbursing the provision and quality of care in relation to the stroke care outcomes. This kind of feedback mechanism is suggested to support healthcare professionals in quality of care and innovation [4, 26, 27]. While NQRs such as Riksstroke can contribute by providing data, it necessitates the feeding of valid data into the system. With Riksstroke, stakeholders across all levels and all regions depicted that a lot of the time set aside for it was consumed by tracking and registering valid data. In total, the time needed for registration of data was vast, leaving limited resources available for healthcare improvement initiatives. Further, the translation of data from the NQR to fellow professionals and managers required stroke care competence in addition to knowledge of what was relevant for the staff and for the unit, in order to nurture ideas and needs for quality improvement. This knowledge was mostly assembled at the politico-administrative level, even though some health professional had accumulated such knowledge and experience of quality improvement. However, the collaboration between the tiers was, as noted, mainly taking place within particular projects. In order for this system to provide learning for an organisation, extended collaboration beyond particular projects is advised [28, 29].

While there are many ways to ‘use’ an NQR in quality improvement [30], the stakeholders across tiers depicted that an NQR such as Riksstroke contributed to an understanding of which aspects to improve in stroke care. Thus, the feedback could contribute to better and safer care, provided that actions were taken in response. The mandate and resources at clinical level varied [15], as well as the engagement at politico-administrative level [16]. Worldwide, undertakings are underway in terms of quality registries or equivalents (for example [31, 32]), and coordination of larger databases is suggested to be beneficial for quality improvement [33]. Meanwhile, our findings illustrate the need to not only secure a standardised input and validity in reported data, but also timely, comprehensible, and accessible feedback – that is, simple but secure pathways to comprehensible data for all key stakeholders [34], along with agreed opportunities for collaboration within a particular context [18]. In our case, this applied to the clinical level, the managerial level as well as the politico-administrative levels of the regions.

In between and across regions and tiers, the leadership engagement differed. Although leadership engagement was described as essential for driving and ensuring quality improvement [35], we found managers and politico-administrative stakeholders who had limited collaboration with the health professionals engaged in stroke care and the local Riksstroke work. Further, although joint stroke process development projects did exist, limited contact beyond these initiatives was described. The lack of regular opportunities to communicate across tiers could mean lost opportunities to collaborative efforts to better understand what and how to improve with regards to stroke care [36]. Further, the lack of information across tiers influences not only the possibilities for the politico-administrative stakeholders to understand but also to use the NQRs and their output [16]. The findings suggest that increased leadership engagement in NQRs is needed [37], to assure a mutual learning across tiers. Further, the need to move beyond registering data to using the output to improve stroke care is evident at all levels of healthcare: at the politico-administrative, hospital and unit levels, and among stroke professionals.

The stroke units and their corresponding regions included in this study represent different experiences in terms of outcomes in the Riksstroke, prior to and at the time of the study. Because these figures could have since changed, a prescription of one way to settle collaboration across tiers with regards to quality improvement using an NQR such as Riksstroke is unsuitable. Further, while the findings represent a range of regions and thus different parts of the country and different types of hospital, an all-inclusive study of users’ experience of Riksstroke in local quality improvement is pending. However, such an investigation has subsequently been done at national level, based on the case study illustrated in this paper. Yet, the experiences of decision-makers and users at politico-administrative and clinical levels can supposedly be transferable to similar settings, within Sweden and elsewhere. The way Swedish healthcare is organised and funded has similarities across the world. Meanwhile, other ways to organise and fund healthcare may invert the opportunity to transfer our findings. However, we suggest that the more general understanding of what hinders and facilitates NQRs, with the Riksstroke as an example, serves to inform quality improvement. This finding may be of interest to a variety of decision-makers, administrators and health professionals engaged in initiating, developing, implementing or using NQRs and similar systems.

## Conclusion

We found the experience of the Riksstroke registry similar across levels and regions. Further, the conditions provided by the outer setting, that is, the national framework, were depicted similarly by the politico-administrative and clinical stakeholders across Sweden. What varied were the regions and stroke unit settings, including whether or not there were opportunities for collaboration with regards to the stroke care processes. The use of the Riksstroke and similar NQRs for setting goals was applied in some regions, in dialogue with the stroke professionals at the clinics, thus providing for multi-level quality improvement. Apart from particular stroke process projects, few opportunities for the continuous sharing of experiences across the levels within the regions existed, but an increased leadership engagement at all levels was needed, along with enhanced understanding of how the NQR can inform stroke care issues and provide for quality improvement.

## Additional file

Additional file 1:
**The details of CFIR and how it was applied in the analysis.** (DOCX 26 kb)
