# Supplementary material for: Depicting the interplay between organisational tiers in the use of a national quality registry to develop quality of care in Sweden
Source: BMC Health Serv Res. 2015 Nov 25;15:519. doi: 10.1186/s12913-015-1188-2 (PMC4660812; doi:10.1186/s12913-015-1188-2)
Supplement: Additional file 1: — The details of CFIR and how it was applied in the analysis. (DOCX 26 kb) [file 12913_2015_1188_MOESM1_ESM.docx]

| Additional file 1. The details of CFIR and how it was applied in the analysis. | | |
| --- | --- | --- |
| **Domain/construct/sub-construct** | **Short definition [12]** | **Definition applied in this study** |
| **I. INTERVENTION CHARACTERISTICS** | | |
| A Intervention Source | Perception of key stakeholders about whether the intervention is externally or internally developed. | How and by whom the registry is developed and whether it is possible to affect the development of the registry |
| B Evidence Strength & Quality | Stakeholders’ perceptions of the quality and validity of evidence supporting the belief that the intervention will have desired outcomes | Validity and reliability of variables in the registry |
| C Relative advantage | Stakeholders’ perception of the advantage of implementing the intervention versus an alternative solution | As in CFIR plus advantages and disadvantages of the intervention (i.e., the registry) itself |
| D Adaptability | The degree to which an intervention can be adapted, tailored, refined, or reinvented to meet local needs | The degree to which the registry as the intervention can be adapted, tailored, refined, or reinvented to meet local needs |
| E Trialability | The ability to test the intervention on a small scale in the organization, and to be able to reverse course (undo implementation) if warranted. | Not applicable |
| F Complexity | Perceived difficulty of implementation, reflected by duration, scope, radicalness, disruptiveness, centrality, and intricacy and number of steps required to implement | How the registry variables are depicted and relate to clinical data, and the process of registering and retrieving data |
| G Design Quality and Packaging | Perceived excellence in how the intervention is bundled, presented, and assembled | How data are registered, presented and accessed by the registry |
| H Cost | Costs of the intervention and costs associated with implementing that intervention including investment, supply, and opportunity costs | As in CFIR, focusing on the local costs for the registry |
| **II. OUTER SETTING** | | |
| A Patient Needs & Resources | The extent to which patient needs, as well as barriers and facilitators to meet those needs are accurately known and prioritized by the organization | To what extent registry data represents patients’ needs and preferences |
| B Cosmopolitanism | The degree to which an organization is networked with other external organizations | Networks and communications outside the region |
| C Peer Pressure | Mimetic or competitive pressure to implement an intervention; typically because most or other key peer or competing organizations have already implemented or in a bid for a competitive edge | As in CFIR, focusing on the registry |
| D External Policy & Incentives | A broad construct that includes external strategies to spread interventions including policy and regulations (governmental or other central entity), external mandates, recommendations and guidelines, pay-for-performance, collaboratives, and public or benchmark reporting | Policy and incentives external to the region, i.e. laws, national clinical guidelines, national benchmark reports and goals set by the national quality registry organisation |
| **III. INNER SETTING** | | |
| A Structural Characteristics | The social architecture, age, maturity, and size of an organization | As in CFIR, focusing on the structures and processes for quality improvement and NQRs at the stroke unit, the hospital and within the region |
| B Networks & Communications | The nature and quality of webs of social networks and the nature and quality of formal and informal communications within an organization | As in CFIR |
| C Culture | Norms, values, and basic assumptions of a given organization | As in CFIR |
| D Implementation Climate | The absorptive capacity for change, shared receptivity of involved individuals to an intervention and the extent to which use of that intervention will be rewarded, supported, and expected within their organization | As in CFIR, focusing on the capacity and extent to which NQRs, particularly Riksstroke, are considered within the organisation (the construct level applied only to meaning units not specific for any of the below sub-constructs) |
| *1 Tension for Change* | The degree to which stakeholders perceive the current situation as intolerable or needing change | As in CFIR |
| *2 Compatibility* | The degree of tangible fit between meaning and values attached to the intervention by involved individuals, how those align with individuals’ own norms, values, and perceived risks and needs, and how the intervention fits with existing workflows and systems | As in CFIR, focusing the stroke units and regions, and particularly, structures and processes for quality improvement and NQRs |
| *3 Relative Priority* | Individuals’ shared perception of the importance of the implementation within the organization | As in CFIR |
| *4 Organizational Incentives & Rewards* | Extrinsic incentives such as goal-sharing awards, performance reviews, promotions, and raises in salary and less tangible incentives such as increased stature or respect | As in CFIR |
| *5 Goals and Feedback* | The degree to which goals are clearly communicated, acted upon, and fed back to staff and alignment of that feedback with goals | As in CFIR |
| *6 Learning Climate* | A climate in which: a) leaders express their own fallibility and need for team members’ assistance and input; b) team members feel that they are essential, valued, and knowledgeable partners in the change process; c) individuals feel psychologically safe to try new methods; and d) there is sufficient time and space for reflective thinking and evaluation | As in CFIR |
| E Readiness for Implementation | Tangible and immediate indicators of organizational commitment to its decision to implement an intervention | Organisational conditions for use of NQR data, particularly from the Riksstroke (the construct level applied only to meaning units not specific for any of the below sub-constructs) |
| *1 Leadership Engagement* | Commitment, involvement, and accountability of leaders and managers with the implementation | As in CFIR |
| *2 Available Resources* | The level of resources dedicated for implementation and on-going operations including money, training, education, physical space, and time | As in CFIR |
| *3 Access to knowledge and information* | Ease of access to digestible information and knowledge about the intervention and how to incorporate it into work tasks | As in CFIR |
| **IV. CHARACTERISTICS OF INDIVIDUALS** | | |
| A Knowledge & Beliefs about the Intervention | Individuals’ attitudes toward and value placed on the intervention as well as familiarity with facts, truths, and principles related to the intervention | Not applicable |
| B Self-efficacy | Individual belief in their own capabilities to execute courses of action to achieve implementation goals | Not applicable |
| C Individual Stage of Change | Characterization of the phase an individual is in, as he or she progresses toward skilled, enthusiastic, and sustained use of the intervention | Not applicable |
| D Individual Identification with Organization | A broad construct related to how individuals perceive the organization and their relationship and degree of commitment with that organization | Not applicable |
| E Other Personal Attributes | A broad construct to include other personal traits such as tolerance of ambiguity, intellectual ability, motivation, values, competence, capacity, and learning style | Not applicable |
| **V. PROCESS** | | |
| A Planning | The degree to which a scheme or method of behaviour and tasks for implementing an intervention are developed in advance and the quality of those schemes or methods | As in CFIR, considering the Riksstroke registry and its (potential) role in quality improvement |
| B Engaging | Attracting and involving appropriate individuals in the implementation and use of the intervention through a combined strategy of social marketing, education, role modelling, training, and other similar activities | As in CFIR, considering the Riksstroke registry and its (potential) role in quality improvement (the construct level applied only to meaning units not specific for any of the below sub-constructs) |
| *1 Opinion Leaders* | Individuals in an organization who have formal or informal influence on the attitudes and beliefs of their colleagues with respect to implementing the intervention | Individuals among staff who are not involved in the Riksstroke and/or quality improvement but hold and voice strong beliefs on these matters, influencing others |
| *2 Formally appointed internal implementation leaders* | Individuals from within the organization who have been formally appointed with responsibility for implementing an intervention as coordinator, project manager, team leader, or other similar role | As in CFIR, considering individuals at clinical and regional level |
| *3 Champions* | “Individuals who dedicate themselves to supporting, marketing, and ‘driving through’ an [implementation]” [101] (p. 182), overcoming indifference or resistance that the intervention may provoke in an organization | As in CFIR |
| *4 External Change Agents* | Individuals who are affiliated with an outside entity who formally influence or facilitate intervention decisions in a desirable direction | As in CFIR and considering those external to the regions |
| C Executing | Carrying out or accomplishing the implementation according to plan | Registration, and retrieval of data in addition to initiating or performing quality improvement based on NQR and/or the Riksstroke data in particular |
| D Reflecting & Evaluating | Quantitative and qualitative feedback about the progress and quality of implementation accompanied with regular personal and team debriefing about progress and experience | Identifying appropriate data in the Riksstroke database, retrieval of data, feedback of data, using the data to understand quality of care or changes made to improve quality of stroke care |
